# CMV-CTL治疗替代供者造血干细胞移植后复发难治性CMV感染17例疗效及安全性

**DOI:** 10.3760/cma.j.issn.0253-2727.2021.10.013

**Published:** 2021-10

**Authors:** 瑞瑞 桂, 珍 李, 璎玲 祖, 娟 王, 燕燕 刘, 兵雷 张, 凤宽 喻, 䶮莉 张, 慧芳 赵, 萍 汪, 永平 宋, 健 周

**Affiliations:** 郑州大学附属肿瘤医院，河南省肿瘤医院血液科 450008 Department of Hematology, Affiliated Cancer Hospital Zhengzhou University, Henan Tumor Hospital, Zhengzhou 450008, China

巨细胞病毒（CMV）感染是异基因造血干细胞移植（allo-HSCT）后常见的严重并发症之一，发生率为37.9％～81.9％[1]–[2]，在替代供者造血干细胞移植（AD-HSCT）后风险更高[3]。尽管更昔洛韦等抗病毒药物可有效治疗CMV感染，但仍有10％患者因CMV感染及其并发症危及生命[4]。近年来allo-HSCT后CMV特异性细胞毒性T淋巴细胞（CMV-CTL）相关细胞免疫治疗取得了一定进展[5]，成为了一种极具潜力的选择。本研究我们回顾性分析2019年7月至2020年10月河南省肿瘤医院CMV-CTL治疗17例AD-HSCT后复发难治性CMV感染患者的临床资料，并评估其疗效和安全性，现报道如下。

## 病例与方法

1. 病例资料：2019年7月至2020年10月我院采用CMV-CTL治疗AD-HSCT后复发难治性CMV感染患者共17例，其中男12例，女5例，中位年龄22（3～53）岁。急性淋巴细胞白血病7例，急性髓系白血病6例，再生障碍性贫血4例。无关供者移植6例，其中HLA 10/10相合4例、9/10相合2例；单倍型移植11例，其中HLA 5/10相合6例、6/10相合3例、8/10相合2例（表1）。

**表1:** 17例异基因造血干细胞移植后接受CMV-CTL治疗CMV感染患者的临床资料

例号	性别	年龄（岁）	诊断	移植类型	HLA相合度	CMV病类型	首次CMV血症距移植时间（d）	外周血CMV-DNA最高水平（×10^3^拷贝数/ml）	输注CTL前末次外周血CMV-DNA水平（×10^3^拷贝数/ml）
1	男	12	ALL	URD-HSCT	9/10	NA	74	11.50	5.46
2	女	8	AA	Haplo-HSCT	8/10	NA	31	34.00	1.26
3	男	31	ALL	Haplo-HSCT	8/10	NA	72	55.00	1.55
4	男	21	AA	URD-HSCT	10/10	NA	40	20.00	4.09
5	男	28	ALL	URD-HSCT	9/10	NA	13	8.13	2.20
6	男	28	AML	Haplo-HSCT	5/10	CMV肺炎	71	15.40	3.16
7	男	40	AML	Haplo-HSCT	6/10	CMV肺炎	28	52.70	
8	女	18	AA	Haplo-HSCT	6/10	CMV肺炎	20	58.70	
9	男	22	ALL	Haplo-HSCT	6/10	CMV视网膜炎	33	4.42	2.20
10	男	28	AA	Haplo-HSCT	5/10	CMV肺炎	33	7.49	
11	男	53	ALL	URD-HSCT	10/10	CMV肺炎	46	15.30	2.63
12	男	24	ALL	Haplo-HSCT	5/10	CMV视网膜炎	38	21.80	
13	男	15	AML	URD-HSCT	10/10	CMV肠炎	68	1500.00	1450.00
14	女	45	AML	Haplo-HSCT	5/10	CMV肺炎	28	670.00	8.80
15	女	3	AML	Haplo-HSCT	5/10	CMV肺炎	34	20.00	4.17
16	女	19	ALL	Haplo-HSCT	5/10	CMV肺炎	39	1140.00	74.50
17	男	15	AML	URD-HSCT	10/10	CMV肺炎	21	515.00	292.00

例号	性别	年龄（岁）	诊断	CTL来源	CTL回输次数	回输距移植时间（d）	回输疗效	CR持续时间（d）	转归
1	男	12	ALL	第三方	1	99	CR	12	无病生存
2	女	8	AA	本人	1	70	CR	3	死于重症肺炎
3	男	31	ALL	本人	1	111	CR	8	无病生存
4	男	21	AA	第三方	1	86	CR	10	无病生存
5	男	28	ALL	第三方	1	36	PR	NA	死于原发病复发
6	男	28	AML	本人	1	103	CR	32	无病生存
7	男	40	AML	本人	1	255	CR	20	无病生存
8	女	18	AA	供者	1	176	CR	15	无病生存
9	男	22	ALL	本人	1	55	CR	10	无病生存
10	男	28	AA	供者	1	310	PR	NA	带病生存
11	男	53	ALL	第三方	1	78	PR	NA	死于TMA
12	男	24	ALL	供者	1	151	PR	NA	带病生存
13	男	15	AML	第三方	1	62	NA	NA	死于脑出血
14	女	45	AML	供者	1	49	NR	NA	带病生存
15	女	3	AML	供者/供者	2	52/78	PR/NR	NA	死于CMV肺炎
16	女	19	ALL	供者/本人	2	57/106	PR/NR	NA	死于CMV肺炎ARDS
17	男	15	AML	第三方/第三方	2	38/63	PR/NR	NA	死于CMV肺炎ARDS

注：CMV：巨细胞病毒；CTL：特异性细胞毒性T淋巴细胞；ALL：急性淋巴细胞白血病；AA：再生障碍性贫血；AML：急性髓系白血病；URD-HSCT：无关供者移植；Haplo-HSCT：单倍型移植；NA：不适用；CR：完全缓解；PR：部分缓解；TMA：血栓性微血管病；ARDS：急性呼吸窘迫综合征

2. 移植物抗宿主病（GVHD）预防：均在+3、+4 d给予环磷酰胺，+5 d始给予环孢素A/他克莫司+霉酚酸酯预防GVHD。

3. 移植后CMV感染及相关定义：CMV血症：连续两次外周血CMV-DNA≥1000拷贝数/ml。早期CMV感染：移植后100 d内发生的CMV感染。晚期CMV感染：移植100 d以后发生的CMV感染。复发性CMV感染：CMV-DNA转阴≥28 d再次检测到CMV-DNA阳性。难治性CMV感染：经正规抗病毒治疗14 d后，CMV-DNA仍上升或进展至CMV病。CMV病：在相关液体和组织样本中发现CMV感染的同时出现终末器官疾病相关的体征和症状。

4. CMV感染的预防和监测：移植前所有供、受者均检测CMV血清抗体或CMV-DNA定量。预处理期间予静脉注射更昔洛韦（5 mg/kg，每日2次）预防CMV感染。造血重建后改用静脉注射阿昔洛韦（10 mg/kg，每日2次），移植1个月后改为口服预防。造血重建至移植后100 d每周至少采用定量PCR检测1次外周血CMV-DNA定量。一旦出现CMV血症，每周检测2次，直至CMV-DNA连续2次转阴后减为每周1次。移植100 d后每2～4周检测1次，直至停用免疫抑制剂。

5. CMV感染的治疗：一旦出现CMV血症，将阿昔洛韦换为更昔洛韦或膦甲酸钠（60 mg/kg，每日2次）直至外周血CMV-DNA转阴，血象不稳定者［ANC≤0.5×10^9^/L和（或）PLT≤20×10^9^/L］首选膦甲酸钠。有条件的患者给予适当减量免疫抑制剂、静脉应用丙种球蛋白或高效价CMV人免疫球蛋白治疗。

6. CMV-CTL的制备：CMV-CTL制备前筛选标准：①难治或复发性CMV血症和CMV病患者，应用更昔洛韦或膦甲酸钠无效时即可开始制备CMV-CTL；②供者CMV-IgG阳性；③预计存活时间3个月以上；④签署CMV-CTL制备知情同意书。采取CMV血清阳性的供者或第三方直系亲属外周血50 ml或患者本人外周血80 ml并分离其中的外周血单核细胞，具体制备流程见文献[6]，由北京赛傲生物技术有限公司制备。

7. CMV-CTL的输注方法和不良反应观察：CMV-CTL的输注标准：①应用更昔洛韦或膦甲酸钠无效，或两者联合也无效；②获得CMV-CTL时外周血CMV-DNA未转阴或进展至CMV病；③非活动性急性GVHD；④签署CMV-CTL输注知情同意书。CMV-CTL的输注方法：第1个疗程：首次输注后部分缓解（PR）或无效（NR）的患者，1～2周后若无GVHD，可重复输注1次，2次间隔1～3周，距首次输注6周仍无效视为治疗失败。第2个疗程：适用于第1个疗程获得反应后复发或进展至CMV病的患者，且CMV-CTL可再次获得，输注方法同第1个疗程。通过外周静脉输注，输注前给予10％葡萄糖酸钙或马来酸氯苯那敏预防过敏反应。输注过程中监测患者生命体征，观察有无瘙痒、荨麻疹、寒战、发热等症状。

8. 疗效判定：CMV-CTL输注疗效分为：①完全缓解（CR）：CMV血症患者外周血连续2次CMV-DNA转阴；CMV病患者累及脏器的相关体征、症状和影像学改变等完全消失。②PR：CMV血症患者外周血CMV-DNA未转阴但拷贝数较输注前至少降低1个log；CMV病患者累及脏器相关体征、症状和影像学改变等好转≥50％。③NR：CMV血症患者外周血CMV-DNA下降未达1个log或持续上升，或进展至CMV病；CMV病患者累及脏器相关体征、症状和影像学改变等无好转或持续进展。有效为CR+PR。

9. 随访：随访起点为首次输注CMV-CTL时间，并记录为0 d。随访截至2020年10月31日，死亡患者随访至死亡时间。

## 结果

1. CMV感染情况：17例CMV感染患者中，CMV血症5例，CMV病12例（CMV肺炎9例、CMV视网膜炎2例、CMV肠炎1例）。首次CMV血症出现的中位时间为移植后34（13～74）d，外周血CMV-DNA最高水平的中位数为21.80（4.42～1500.00）×10^3^拷贝数/ml。共13例患者输注CMV-CTL前末次外周血CMV-DNA阳性，中位数为4.09（1.26～1450.00）×10^3^拷贝数/ml（表1）。

2. CMV-CTL输注情况：7例次CMV-CTL来自供者，7例次来自第三方直系亲属，6例次来自患者本人。首次输注CMV-CTL时间为移植后78（36～310）d。单次输注的CD8^+^细胞中位数为97.5％（90.9％～98.8％），中位细胞活率为99.3％（95.8％～99.9％），中位CMV-CTL细胞数为7.80（0.60～17.90）×10^8^，中位输注量为1.31（0.30～2.75）×10^7^/kg。

3. CMV-CTL疗效评估：5例CMV血症患者均仅接受1个疗程CMV-CTL输注，4例CR，1例PR，CR的中位时间为输注后9（3～12）d。12例CMV病患者中，9例患者仅接受1个疗程CMV-CTL输注，4例CR，3例PR，1例NR，1例患者因在输注后第3天死于脑出血无法判定疗效。3例患者接受了2个疗程CMV-CTL治疗，首次输注后均获得PR，但后来分别在输注后15、30、11 d进展为CMV肺炎，故给予第2次CMV-CTL治疗，但均治疗无效。第2次CMV-CTL治疗中，1例患者因缺乏供者，更换为患者本人来源的CMV-CTL，余2例CMV-CTL来源不变。17例患者CMV-CTL治疗总有效率（ORR）为78.95％，CR的中位时间为输注后11（3～32）d（表1）。

4. 转归：随访至HSCT后1年，共7例患者死亡，中位死亡时间为输注后63（3～148）d。其中3例死于CMV肺炎，1例死于肺侵袭性真菌病，1例死于脑出血，1例死于血栓性微血管病（TMA），1例死于原发病复发（表1）。

5. 安全性评估：共输注20例次CMV-CTL，仅1例次（例2）输注后出现过敏性休克，予积极抗过敏后控制，余19例次输注过程顺利，未出现不适症状。所有患者输注后均未观察到GVHD症状。

## 讨论

CMV感染是AD-HSCT后患者主要死亡原因之一，目前以更昔洛韦为首的抗病毒治疗仍然是一线治疗手段[7]。但更昔洛韦相关的骨髓抑制等毒性显著，可直接影响CTL的产生，延缓CTL相关的免疫重建，增加晚期CMV感染风险，在缺乏细胞免疫治疗的情况下，其疗效有限[8]。细胞免疫治疗包括供者淋巴细胞输注（DLI）和CMV-CTL，DLI会增加GVHD和晚期CMV感染风险[9]，而CMV-CTL过继免疫治疗可通过提高受者体内CMV特异性T细胞的数量和功能，重建CMV特异性抗病毒免疫作用，从而提高受者对CMV感染或晚期CMV再激活的防御能力[6]。

清髓性或去T细胞的预处理、供者CMV抗体阴性、移植后早期更昔洛韦的应用、抗GVHD相关的免疫抑制治疗均可延缓移植后CTL相关免疫的重建[10]。AD-HSCT患者因CTL重建延迟，导致CMV再激活风险明显增高，尤其是抗病毒药物治疗无效的复发难治性CMV感染[11]。allo-HSCT后预防性输注供者来源CMV-CTL的ORR接近100％，对已发生CMV感染患者进行抢先治疗的ORR为70％～85％[11]。即使在复发难治性CMV感染患者中，CMV-CTL仍具有较高的有效率。徐郑丽等[12]研究证实allo-HSCT后复发难治性CMV感染患者在接受CMV-CTL治疗后，89.30％的患者获得CR。Feuchtinger等[13]报道CMV-CTL治疗复发难治性CMV感染患者的ORR为83.30％。本研究中的CMV-CTL治疗ORR为78.95％，略低于上述文献报道数据，可能原因是我们的研究对象为AD-HSCT患者，且CMV病患者比例高于其他文献资料，抗CMV感染治疗难度大。

CMV-CTL治疗CMV血症患者疗效优于CMV病患者。5例CMV血症患者CR率为80％，ORR为100％，而12例CMV病患者CR率为28.57％，ORR为71.43％，提示在药物抗病毒治疗效果不佳且尚未进展至CMV病时尽早输注CMV-CTL可能获得更好疗效。Blyth等[14]证实，早期输注CMV-CTL组抗病毒药物应用的总量和时间均明显减少（*P*＝0.03），且无晚期CMV再激活。这提示移植后早期给予CMV-CTL降低CMV病的发生率和晚期CMV再激活风险的同时，可降低对抗病毒药物治疗的需求，从而降低抗病毒药物相关毒性和经济负担。其原因可能是早期输注CMV-CTL可促进重建即时和长期预防CMV免疫功能，保护患者安全度过移植后免疫抑制阶段，直到受者免疫重建。

单次输注的CMV-CTL抗病毒作用不持久，为保证疗效，必要时可再次输注CMV-CTL，其影响因素可能有：

（1）输注的CMV-CTL细胞数量和纯度。目前报道的各中心单次输注的CMV-CTL细胞数量不一致，尚无相关指南推荐，最佳的单次细胞数量应在满足最大疗效的同时，尽可能降低GVHD等不良反应发生率。目前报道的均是单次输注的CMV-CTL绝对值，笔者以为，患者每公斤体重输注的CMV-CTL数量是更合适的衡量标准。我们单次输注CMV-CTL细胞中位数为7.80（0.60～17.90）×10^8^，按患者每公斤体重计算为1.31（0.30～2.75）×10^7^/kg，明显高于其他中心，但无一例发生GVHD。考虑为特异性CTL的检测手段不同，我们采用检测IFN-γ表达来确定特异性CTL数量，但因为多种细胞均可分泌IFN，且有胞内和胞外之分，所以结果并不精确，需要进一步探索出更精准的检测手段。

（2）输注前CMV的负荷量。这和受者免疫功能受损程度有关，移植后早期获得CMV-CTL免疫重建的患者发生CMV相关并发症的风险降低[15]–[16]，而高CMV-DNA水平与高风险持续性CMV血症、CMV病有关[17]。本研究也证实了这一点，5例CMV血症患者首次发生CMV血症的中位时间为移植后40（13～74）d，输注前最高CMV-DNA水平的中位数是20.00（8.13～55.00）×10^3^拷贝数/ml；12例CMV病患者首次出现CMV血症的中位时间为33（20～71）d，输注前最高CMV-DNA水平的中位数是37.25（4.42～1500.00）×10^3^拷贝数/ml。已证实药物抗病毒治疗联合CMV-CTL过继免疫治疗作为一线治疗相结合，可显著降低持续性CMV感染和CMV病的风险[18]。但CMV-CTL制备周期长，至少需要2周左右，易错失最佳治疗时机。所以针对高危的复发难治性CMV感染患者，可考虑尽早培养CMV-CTL。

CMV-CTL最常见的不良反应是可诱发GVHD，但可以通过控制CMV-CTL细胞数量和纯度降低其风险。Mackinnon等[19]证实CMV-CTL在体内扩增3～5倍对数级能有效抑制病毒复制而不增加GVHD风险。本研究中未发生CMV-CTL相关GVHD事件，除了65％的CMV-CTL来自原供者和患者本人这一因素外，可能和我们应用的PT/CY预防GVHD方案有关，此方案已被证实可显著降低AD-HSCT后急、慢性GVHD风险[20]。此外，因样本量有限，目前并未发现不同来源的CMV-CTL在疗效方面的优劣，需要扩大数据进一步证实。

综上所述，CMV-CTL过继免疫治疗是AD-HSCT后复发难治性CMV感染的一种安全、有效的治疗措施。建议在药物抗病毒治疗效果不佳且尚未进展至CMV病时尽早输注CMV-CTL。
